# Q Fever in Thailand

**DOI:** 10.3201/eid0909.030086

**Published:** 2003-09

**Authors:** Yupin Suputtamongkol, Jean-Marc Rolain, Kitti Losuwanaruk, Kanigar Niwatayakul, Chuanpit Suthinont, Wirongrong Chierakul, Kriangsak Pimda, Didier Raoult

**Affiliations:** * Mahidol University, Bangkok, Thailand; † Faculté de Médecine, Marseille, France; ‡ Banmai Chaiyapod Hospital, Bureerum Province, Thailand; § Maharat Nakornrachasema Hospital, Nakornrachasema Province, Thailand; ¶ Loei Hospital, Loei Province, Thailand; # Udornthani Hospital, Udornthani Province, Thailand

**Keywords:** Acute Q fever, Thailand

**To the Editor:** *Coxiella burnetii,* a strict intracellular bacterium, is the etiologic agent of Q fever, a worldwide zoonosis. Humans are infected by inhaling contaminated aerosols from amniotic fluid or placenta or handling contaminated wool (*1*). The bacterium is highly infectious by the aerosol route. Two forms of the disease are typical: acute and chronic. Acute Q fever is the primary infection and in specific hosts may become chronic (*1*,*2*). The major clinical manifestations of acute Q fever are pneumonia and hepatitis. Less common clinical manifestations are aseptic meningitis and/or encephalitis, pancreatitis, lymphadenopathy that mimics lymphoma, erythrema nodosum, bone marrow necrosis, hemolytic anemia, and splenic rupture (*2*). The main clinical manifestation of the chronic form is culture-negative endocarditis, but infection of vascular grafts or aneurysms, hepatitis, osteomyelitis, and prolonged fever have also been described (*1*,*2*). Fluoroquinolones, co-trimoxazole, and doxycycline are active against C*. burnetii* in vitro, and ceftriaxone has been shown to have a bacteriostatic effect and could be effective in the phagolysosome of *C. burnetii*–infected cells (*3*). However, the treatment of choice for Q fever is doxycycline.

The incidence of this disease is largely unknown, especially in Asia. Q fever has been reported from Japan and China (*1*). Seroepidemiologic surveys have shown that subclinical infection is common worldwide. Large outbreaks of Q fever have also been reported in many countries in Europe (*4*). A case series of acute Q fever was diagnosed in a prospective study in patients with acute febrile illness who were admitted to four hospitals in northeastern Thailand: Udornthani Hospital, Udornthani Province; Maharat Nakornrachasema Hospital, Nakornrachasema Province; Loei Hospital, Loei Province; and Banmai Chaiyapod Hospital, Bureerum Province. Two serum samples were taken from these patients, on admission and at a 2- to 4-week outpatient follow-up visit, and stored at –20°C until serologic tests were performed at the Faculty of Medicine Siriraj Hospital, Mahidol University, and the National Research Institute of Health, Public Health Ministry of Thailand. All serum samples were tested for the serologic diagnosis of leptospirosis, scrub typhus, murine typhus, and dengue infection as previously described (*5*,*6*). After these serologic tests were performed, serum samples from patients with unknown diagnosis were sent for the serologic test for Q fever at Unité des Rickettsies, Faculté de Médecine, Marseille, France. The microimmunofluorescent antibody test, using a panel antigen of *C. burnetii, Rickettsial honei, R. helvetica, R. japonica, R. felis, R. typhi, Bartonella henselae, B. quintana, Anaplasma phagocytophila,* and *Orientia tsutsugamushi*, was used as described previously (*6*).

A total of 1,171 serum specimens from 678 patients were tested for Q fever. Nine case-patients (1.3%, eight male and one female) fulfilled the diagnosis of acute Q fever. The median age was 42 (range 15–62) years. All patients were rice farmers, and their farm animals were chicken and cattle. The median duration of fever was 3 (range 1–7) days before admission into the hospital. When initially seen, all patients had acute febrile illness, headache, and generalized myalgia (i.e., a flulike syndrome). Clinical manifestations of acute Q fever in these patients ranged from this flulike syndrome (three patients), pneumonitis (one patient), hepatitis (two patients), pneumonitis and renal dysfunction (one patient), hepatitis and renal dysfunction (one patient), to severe myocarditis and acute renal failure (one patient). An epidemic of leptospirosis has been occurring in Thailand since 1996 (*7*). All patients in this study received a diagnosis of either leptospirosis or acute fever of undefined cause; therefore, empirical therapy, including penicillin G sodium, doxycycline, and cefotaxime or ceftriaxone, was administered. The patient with hepatic and renal dysfunction was treated with co-trimoxazole. The patient who had severe myocarditis and acute renal failure was treated with a penicillin G sodium and doxycycline combination. He also received a dopamine infusion and hemodialysis. The median duration between admission and a reduction of fever was 3 days (range 1–7) in this case series.

Results of several seroprevalence studies, using the complement fixation test, conducted in both humans and animals suggest that *C. burnetii* infection has been widespread in Thailand since 1966 (*8*). The prevalence in asymptomatic persons varies from 0.4% to 2.6% (*9*), and studies in domestic animals show that the highest prevalence of this infection occurs in dogs (28.1%). The prevalence in goats, sheep, and cattle varies from 2.3% to 6.1% (*9*). However, this clinical case series of acute Q fever is the first diagnosed in this country. The disease was diagnosed in patients in four hospitals, situated in various parts of the northeastern region of Thailand. These data confirmed that Q fever is widespread in this country. The disease had been unrecognized previously because the specific serologic test was not widely available in Thailand.

A self-limited course was suspected in four cases in this series. However, severe cases, especially those with myocarditis, could be fatal. Therefore, doxycycline should be an empirical therapy for patients with acute febrile illness in areas where leptospirosis, scrub typhus, and acute Q fever are suspected, such as in rural Thailand. Further studies to investigate the epidemiology of Q fever in this country are needed.
